# Efficacy of first‐line dual oral pyrotinib plus capetabine therapy in HER2‐positive metastatic breast cancer: A real‐world retrospective study

**DOI:** 10.1002/cam4.7256

**Published:** 2024-05-29

**Authors:** Shuang Dai, Yong Zhang, Xiang Tan, Feng Luo, Xi Yan

**Affiliations:** ^1^ Department of Medical Oncology, Cancer Center, West China Hospital Sichuan University Chengdu Sichuan China; ^2^ Lung Cancer Center, West China Hospital Sichuan University Chengdu Sichuan China; ^3^ Department of Thoracic Surgery The First Affiliated Hospital of Guangxi Medical University Nanning Guangxi China; ^4^ Breast Center, West China Hospital Sichuan University Chengdu Sichuan China

**Keywords:** breast cancer, HER2, metastasis, pyrotinib

## Abstract

**Background:**

The combination of dual‐targeted human epidermal growth factor receptor 2 (HER2) therapy and chemotherapy is the standard first‐line regimen for recurrent/metastatic breast cancer (mBC). However, the toxicity of such combination therapy can lead to some patients being unable to tolerate adverse events or bear treatment costs. As a novel irreversible pan‐ErbB receptor TKI (pyrotinib), can the dual oral administration of pyrotinib plus capetabine (PyroC) provide first‐line survival benefits and serve as a more affordable treatment option?

**Methods:**

This real‐world retrospective study included patients diagnosed with HER2‐positive mBC who received PyroC as a first‐line treatment at West China Hospital between May 2018 and July 2023. The survival data and toxicity profiles were reported in this study.

**Results:**

A total of 64 patients received PyroC as first‐line therapy. The median progression‐free survival (PFS) was 19.6 months (95% CI 15.0–27.2), while overall survival (OS) has not yet been reached. Kaplan–Meier analysis indicated that age (≥60, *p* = 0.03) and metastasis sites (*p* = 0.004) were related to poor efficacy of PyroC, while there was no relationship between effectiveness and menstrual status, hormone receptor (HR) status or previous treatment with anti‐HER2 therapy. Furthermore, the objective response rate (ORR) and disease control rate (DCR) were 79.7% and 98.4%, respectively. Of the patients, 78.1% reported treatment‐related adverse events (TRAEs). The predominant adverse events were diarrhea (*n* = 46, 71.9%) and hand‐foot syndrome (*n* = 10, 15.6%).

**Conclusion:**

The dual oral administration regimen (PyroC) has a promising ORR or PFS in HER2‐positive mBC patients, with an acceptable safety profile and convenience.

## 1 INTRODUCTION

Breast cancer patients express the human epidermal growth factor receptor 2 (HER2 or ERBB2) in around 15%–20% of the cases,^1^ which generally present high invasiveness and are associated with poor prognosis.^2^ Over the last decade, with the arrival of HER‐2 targeted drugs, there has been a significant survival benefit for HER2‐positive locally recurrent or metastatic BC (mBC) patients in clinical trials, regardless of the HR status.^3^, ^4^, ^5^ Obviously, in comparison to single chemotherapy, the use of trastuzumab has been shown to extend the overall survival (OS) of mBC patients by 5–8 months.^6^ Additionally, according to the CLEOPATRA trial, double blockade (trastuzumab and pertuzumab) plus chemotherapy can further improve the median OS from 40.8 to 57.1 months in the control arm (trastuzumab plus chemotherapy).^7^ HER2‐targeted therapy combined with chemotherapy is recommended as the first‐line treatment for HER2‐positive mBC. To date, there are three categories of HER‐2 targeted drugs: monoclonal antibodies (trastuzumab and pertuzumab),^8^ tyrosine kinase inhibitors (lapatinib, neratinib, and tucatinib),^5^, ^8^, ^9^, ^10^ and antibody‐drug conjugates (ado‐trastuzumab emtansine [T‐DM1] and trastuzumab deruxtecan [DS8201]).^3^, ^11^ Despite this, patients can still develop resistance to anti‐HER2 treatment mentioned above, signifying the need for supplementary treatment strategies for mBC.

Pyrotinib (Pyro) is a small oral molecule, irreversible tyrosine kinase inhibitor that was developed independently in China.^12^ Pyrotinib plus capecitabine (PyroC) has been regarded as a second‐line treatment for HER2‐positive mBC. In an open‐label, multicenter, randomized, phase II study,^13^ PyroC exhibited a notable objective response rate (ORR) of 78.5% compared to the control group receiving lapatinib plus capecitabine (57.1%), with or without previous trastuzumab treatment. Moreover, the phase III PHOEBE study also confirmed that PyroC could significantly prolong median progression‐free survival (mPFS), surpassing the efficacy of lapatinib plus capecitabine (12.5 months vs. 6.8 months) in second‐line therapy for HER2‐positive mBC.^14^ In particular, for patients with brain metastases, based on a phase II PERMEATE study,^15^ PyroC significantly improved the intracranial response rate (CNS‐ORR) (74.6% vs. 65.9%) and mPFS (11.3 months vs. 5.5 months) compared with those for lapatinib plus capecitabine in LANDSCAPE study.^16^ However, evidence of pyrotinib for first‐line treatments is limited. At present, researchers are investigating different combination regimens incorporating pyrotinib to continuously enhance its evidence‐based effectiveness as a first‐line treatment for mBC. With reference to pertuzumab combined with trastuzumab (H) and docetaxel (T), the standard first‐line dual anti‐HER2 therapy for HER2‐positive mBC, PHILA trial proposed a new treatment strategy termed PyroHT (Pyro combined with HT).^17^ This study found that PyroHT can significantly prolong the PFS of patients to 24.3 months, compared to 18.7 months of CLEOPATRA trial, offering a new effective option for first‐line treatment of HER2‐positive advanced BC. Furthermore, the single‐arm, multicenter phase II PANDORA study also examined the effectiveness of combining Pyro with docetaxel as a first‐line treatment for HER2‐positive mBC. The study enrolled 79 patients, and the mPFS was 15.97 months. In a phase II PLEHERM study, the combination of Pyro and letrozole as first‐line treatment for 53 patients with HR^+^/HER2^+^ mBC demonstrated favorable anti‐tumor effects. The ORR was 64.2%, and the mPFS was 13.7 months.^18^ Despite the substantial survival benefits, the new regimen still faces a common dilemma: the dual‐targeted therapy combined with intravenous chemotherapy has increased toxicity, making it difficult for some patients to tolerate adverse reactions or bear treatment costs. Here comes the question: can the dual oral second‐line PyroC regimen be used as first‐line treatment for HER2‐positive mBC, and how about its efficacy and safety?

Some retrospective studies, which enrolled patients partially receiving PyroC therapy, have shown improvement in survival with the use of Pyro‐based regimens as first‐line treatment for HER2‐positive mBC.^19^, ^20^ However, the evidence level is not convincing enough. For instance, Yin et al. reported that mPFS was 20.9 months for patients receiving first‐line Pyro treatment, which enrolled only 28 patients, partially treated by PyroC.^20^ A single‐arm, multicenter, phase II PICTURE study was published on August 9, 2023. It enrolled 49 patients who were resistant to trastuzumab and received PyroC. The study revealed a mPFS of 17.8 months,^21^ providing more beneficial options for first‐line treatment of mBC. However, more data is needed to verify the efficacy of PyroC, especially in the real world. Here, we performed a retrospective analysis to preliminarily assess the efficacy and safety of PyroC as a first‐line treatment for HER2‐positive mBC in the real world based on the 64 patients treated with PyroC at West China Hospital of Sichuan University in China. It is expected to enrich the evidence for PyroC as an alternative to first‐line treatment for HER2‐positive mBC and have guiding implications in the treatment/research of HER2‐positive mBC for clinicians and scientists in the future.

## 2 MATERIALS AND METHODS

### 2.1 Study design and data collection

A retrospective, observational cohort study was conducted at the West China Hospital of Sichuan University in China. The study was approved by the Biomedical Research Committee (Approval No. 2020876) and was conducted in accordance with the Declaration of Helsinki. It has also been approved by the Ethics Committee of West China Hospital, Sichuan University. All participants provided written informed consent prior to enrollment.

### 2.2 Patients' eligibility

The inclusion criteria were as follows: (1) newly diagnosed HER2‐positive breast cancer patients who are diagnosed with locally advanced unresectable disease and stage IV disease with primary resistance to trastuzumab or with poor tolerability to intravenous treatments. Patients who had received (neo)adjuvant trastuzumab‐based therapy and had recurrence beyond 12 months from its completion are eligible. The definition of HER2 positivity involved an immunohistochemistry score of 3+ or fluorescence in situ hybridization (FISH) score of + in either primary or metastatic tumor tissue; (2) patients aged between 18 and 80 years; (3) at least one measurable lesion according to the Response Evaluation Criteria in Solid Tumors (RECIST 1.1); (4) an Eastern Cooperative Oncology Group (ECOG) performance status of 0–2; (5) an expected survival of more than 12 weeks; and (6) normal organ function, including blood tests for liver and renal function prior to chemotherapy, with no contraindications for treatment.

### 2.3 Treatment administration

Pyrotinib (Irenia, Jiangsu Hengrui Medicine Co., Ltd.) are a small oral molecule for HER2‐positive breast cancer. All metastatic or local recurrent patients were treated with PyroC, which involved oral administration of 1000 mg/m^2^ capecitabine twice daily between Days 1 and 14, along with Pyro at a dosage of 400 mg/day (if not tolerated, the dose can be adjusted to 320 mg/day). The patients receiving PyroC were assessed for treatment efficacy after every two cycles of treatment. Treatment is continued until disease progression (PD) or intolerance to the treatment occurs.

### 2.4 Outcomes

The main focus of this study is to evaluate PFS as the primary endpoint. PFS is measured from the initiation of treatment until the earliest instance of any event or death resulting from any cause. Secondary endpoints include OS, ORR, and disease control rate (DCR). Evaluation of treatment response by computed tomography (CT) was performed after the first 4 weeks. ORR is the proportion of participants with the best result of CR plus PR, and DCR is calculated as CR plus PR plus SD.

All measurable lesions were measured at baseline before treatment and were regularly monitored by CT and magnetic resonance imaging at 6‐week intervals. Response and progression were assessed by the experienced treating physician according to RECIST 1.1. Physicians provided patients with a card to record any observed side effects after the first dose. The information recorded on the card will be used to document the patient's medical record at subsequent follow‐up visits. We retrospectively examined the patients' medical records, and collected additional information about adverse events through telephone visits. Adverse events were evaluated according to the National Cancer Institute—Common Terminology Criteria for Adverse Events (NCI‐CTC 4.0).

### 2.5 Statistical analyses

Quantitative data are presented as the median value, whereas the presentation of qualitative and ranked data is in terms of rate and proportion. Survival analysis was completed using the Kaplan–Meier analysis with Brookmeyer–Crowley test. *p* < 0.05 are considered significant. All analyses were conducted using R 4.2.1.

## 3 RESULTS

### 3.1 Baseline patient characteristics

Between May 2018 and January 2023, a total of 64 patients were included. Among them, 13 patients (20.3%) were initially diagnosed with stage IV disease, while the remaining patients (*n* = 51) had early/locally advanced unresectable disease. Among these patients, 16 patients (25%) had received (neo)adjuvant trastuzumab‐based therapy and experienced recurrence more than 12 months after completing it. All patients were treated with PyroC as a first‐line treatment. The demographic and basic characteristics are depicted in Table 1. Overall, the median age was 53 years. Twenty‐one cases (32.8%) were premenopausal, and 43 cases (67.2%) were postmenopausal. Of the total cases, 25 (39.1%) were hormone receptor (HR)‐positive. Among them, 51 cases (79.7%) had visceral metastases. The brain, bone, liver, and lung are the most common metastasis sites. Importantly, 31.2% of patients have at least two metastatic/recurred lesions.

**TABLE 1:** Demographic characteristics of patients who received pyrotinib plus capetabine as a first‐line therapy.

Characteristic	Patients (%) (*N* = 64)
Age at diagnosis (median, years)	53
Menstrual status	
Premenopausal	21 (32.8%)
Postmenopausal	43 (67.2%)
HR status	
Positive	25 (39.1%)
Negative	39 (60.9%)
HER2 status	
IHC 3+	55 (85.9%)
IHC 2+, FISH+	9 (14.1%)
TNM stage at initial diagnosis	
IA	2 (3.1%)
IB	1 (1.6%)
IIA	9 (14.1%)
IIB	16 (25.0%)
IIIA	15 (23.4%)
IIIB	2 (3.1%)
IIIC	6 (9.4%)
IV	13 (20.3%)
Previous treatment with anti‐HER2 therapy	
Yes	52 (81.3%)
No	12 (18.7%)
Visceral metastases	
Yes	51 (79.7%)
No	13 (20.3%)
Metastasis site	
Lymph nodes/single‐site	44 (68.8%)
Single‐site + lymph nodes	12 (18.8%)
Multiple‐site	7
Lung metastasis	
0	45 (70.3%)
1	6 (9.4%)
≥2	13 (20.3%)
Brain metastasis	
0	54 (84.4%)
1	6 (9.4%)
≥2	4 (6.2%)
Bone metastasis	
0	41 (64.0%)
1	14 (21.9%)
≥2	9 (14.1%)

### 3.2 Efficacy outcomes

**FIGURE 1:**
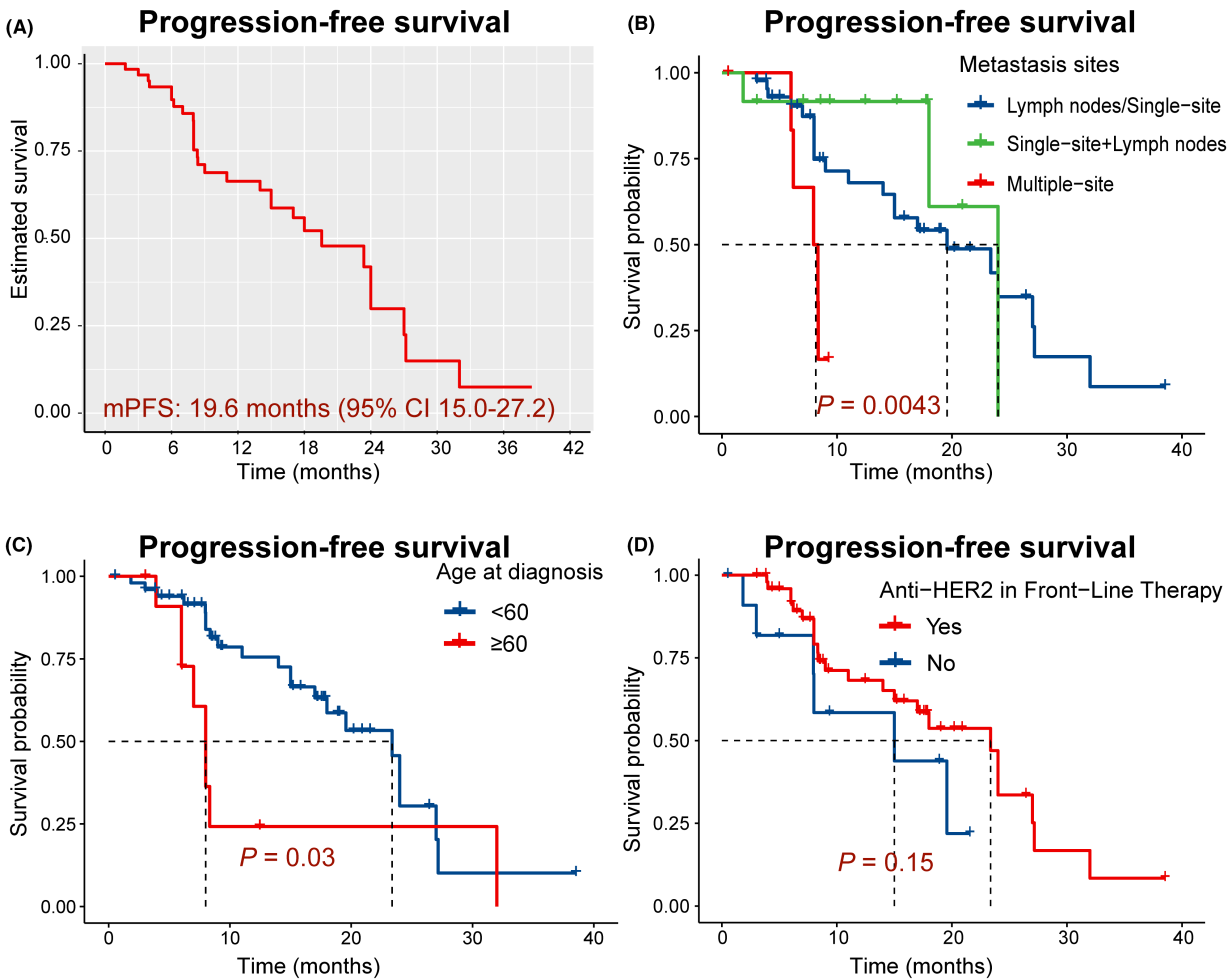
Kaplan–Meier survival analysis. (A) progression‐free survival (PFS) of all patients treated with pyrotinib plus capecitabine (PyroC) as first‐line therapy. (B–D) Kaplan–Meier estimate stratified by metastasis sites, age at diagnosis, and previous use of HER2‐targeted therapy, respectively.

**FIGURE 2:**
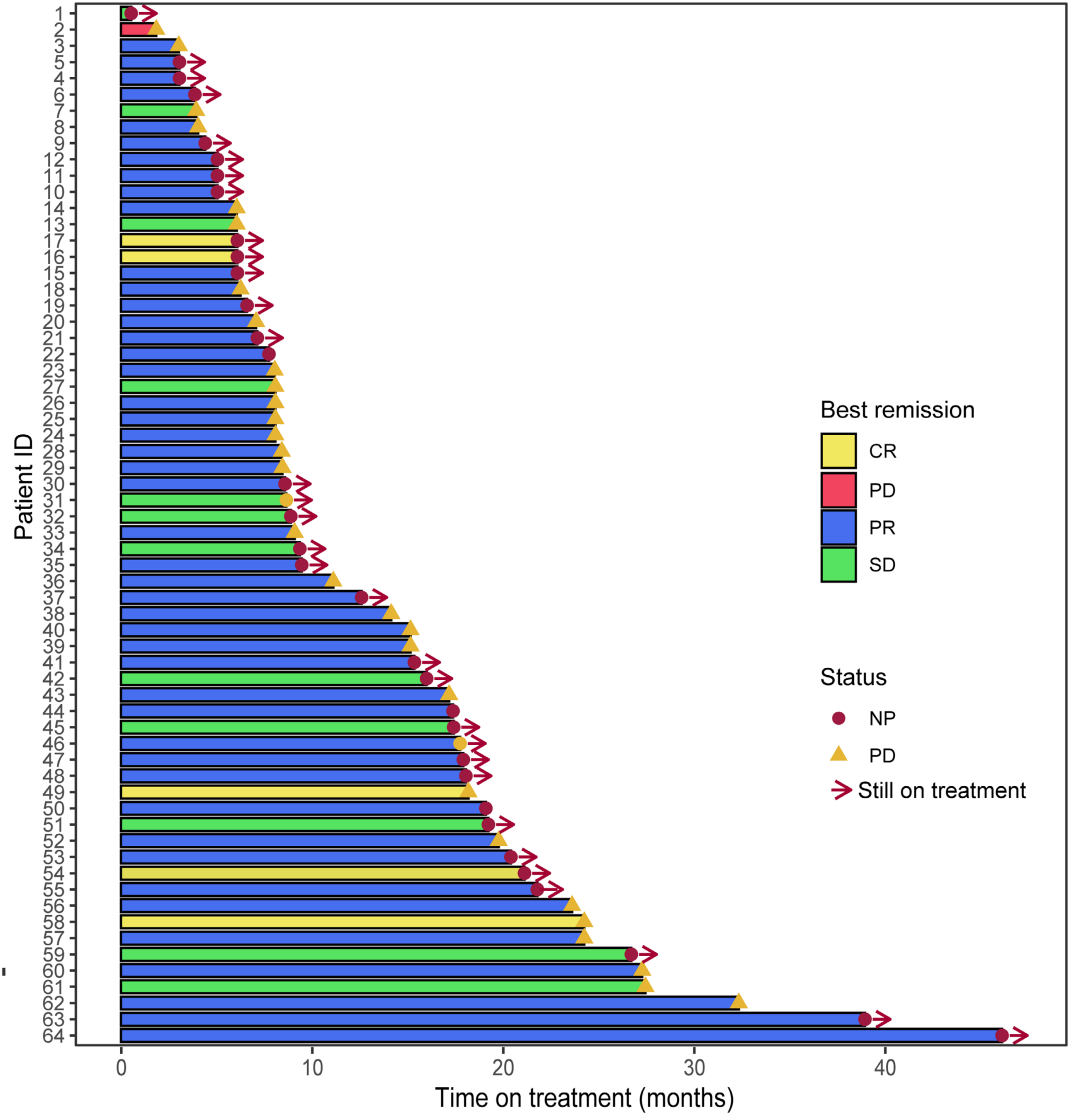
Summary of treatment response to pyrotinib plus capecitabine (PyroC) as first‐line therapy in patients with HER2‐positive metastatic breast cancer.

The median PFS (mPFS) was 19.6 months (95% CI 15.0–27.2) (Figure 1A). However, patients with more than two visceral metastases (*n* = 7) had a shorter mPFS of 8.15 months (95% CI 6.2‐not estimable) (Figure 1B, *p* = 0.004). OS has not reached. We used Kaplan–Meier analysis, which indicated that age (≥60, *n* = 12, *p* = 0.03) and metastasis sites (*p* = 0.004) were related to the poor efficacy of PyroC. However, no correlation was found between PyroC effectiveness and menstrual status, HR status, and anti‐HER2 in front‐line therapy (Figure 1D, *p* >0.05). In addition, the ORR and DCR were 79.7% and 98.4%, respectively. Five patients achieved a CR (Figure 2).

### 3.3 Safety outcomes

78.1% of patients (*n*=50) reported treatment‐related adverse event (TRAE), no treatment‐related serious AEs occurred. The major TRAEs is diarrhea, occurring in 46 cases (71.9%), and 97.8% were grade (G) 1–2, while only one G3/4 diarrhea occurred. Other AEs included hand‐foot syndrome (*n* = 10, 15.6%), and vomiting (*n* = 9, 14.1%). All of AEs are summarized in Table 2.

**TABLE 2:** Adverse events of patients who received pyrotinib as a First‐Line therapy.

Characteristic	Patients (%) (*N* = 64)
Diarrhea	
Yes	46 (71.9%)
No	16 (25.0%)
Diarrhea grade (%)	
I	18 (39.1%)
II	27 (58.7%)
III	1 (2.2%)
Hand‐foot syndrome	
Yes	10 (15.6%)
No	54 (84.4%)
Hand‐foot syndrome grade (%)	
I	6 (60%)
II	3 (30%)
III	1 (10%)
Vomiting	
Yes	9 (14.1%)
No	54 (84.4)

## 4 DISCUSSION

Undoubtedly, clinical trials are a potent scientific tool for evaluating the safety and efficacy of new drugs. Nevertheless, real‐world studies have the potential to fill knowledge gaps and have noteworthy implications in the development of clinical trials to some extent. In this study, we examined a cohort of 64 HER2‐positive mBC patients who received dual oral PyroC regimen as first‐line therapy. Our results indicate that the mPFS was 19.6 months, with an ORR of 79.7%. Patients who were elderly or had multiple metastasis sites had a poor prognosis. However, the efficacy of PyroC was found to be independent of menstrual status, HR status, previous treatment with anti‐HER2 therapy, or metastasis type. Although half of patients reported TRAEs, no treatment‐related serious AEs occurred.

Trastuzumab plus pertuzumab (HP) dual‐target therapy has been the standard of care in HER2^+^ breast cancer, with an OS of 57.1 months.^7^ However, despite the availability of dual‐target therapy, some patients continue to experience primary or secondary resistance to this therapy.^7^, ^22^ Furthermore, the concern of economic status and drug tolerance in the treatment of mBC patients also requires much attention. Hence, there is a crucial need for another efficacious alternative and affordable anti‐HER2 regimen. To date, tremendous attention has been given to Pyro‐based regimens as an alternative first‐line treatment option for HER2‐positive mBC.^14^, ^17^, ^23^, ^24^ Compared with other studies,^17^, ^24^ the population included in this study was mainly those with insufficient financial means to cover the costs associated with dual‐targeted therapies and those unwilling to or unsuitable for receiving intravenous infusion therapy. PyroC is characterized by its oral administration and affordability, so it offers a new option for patients with underdeveloped economy and poor tolerance of intravenous treatments. In terms of survival, we also found that PyroC provide a noninferior treatment outcome (mPFS: 19.6 months, ORR: 79.7%), consistent with PHILA study (mPFS: 24.3 months).^17^ We expected that more reliable first‐line survival data will provide strong evidence for the future use of PyroC.

Of note, treatment response for patients with ≥60 years of age and multiple visceral metastatic sites was relatively poor. The former is probably because patients have poorer tolerance, while the latter means a greater chance of visceral crises.^25^ Patients with visceral crisis require rapidly effective treatment to address severe organ dysfunction,^26^ but adequate assessment of randomized trials on these existing anti‐HER2‐targeted therapies' effectiveness has not yet been conducted. Currently, only case reports with weak levels of evidence have shown promising anti‐tumor effects for patients with visceral crises when combining HER2‐targeted therapy with chemotherapy.^27^ Therefore, the combination of pyrotinib‐based therapies (including PyroC) needs to be further validated in dealing with a visceral crisis. Furthermore, we found that the efficacy of pyrotinib is not affected by the use of HER2‐targeted therapy as front‐line treatment including both neoadjuvant and adjuvant settings.

In terms of the toxicity of PyroC, we can see that adverse effects symptoms are manageable. The major TRAEs is diarrhea, occurring in 74.2%, and 96.9% were Grade 1–2. Compared with PHILA^17^ and PHENIX^28^ study, our study did not observe hematologic adverse effects and had lower incidence of adverse effects such as diarrhea. Of course, the first concern is the cumulative side effects of the triple combination therapy in PHILA and PHENIX studies. In addition, all patients we included were first‐line users with better ECOG scores and better tolerability.

Several limitations deserve mention. Firstly, our study may lead to selection bias using retrospective data. Secondly, the sample of study is relatively small and single‐center. Thirdly, this study lacks information on long‐term OS. In‐depth studies are needed to validate OS of switching TKI treatment. Nevertheless, the findings of our study still have noteworthy real‐world survival outcomes for PyroC in HER2^+^ mBC patients, suggesting that dual oral PyroC regimen may be a supplementary strategy as first‐line treatment.

## 5 CONCLUSIONS

Dual oral PyroC regimen represents a promising therapeutic option as first‐line therapy for patients with HER2‐positive mBC, with an acceptable safety profile, affordability, and convenience.

## AUTHOR CONTRIBUTIONS

**Shuang Dai:** Formal analysis (equal); investigation (equal); supervision (equal); validation (equal); visualization (equal); writing – original draft (equal). **Yong Zhang:** Formal analysis (equal); investigation (equal); methodology (equal); validation (equal); visualization (equal); writing – original draft (equal). **Xiang Tan:** Data curation (equal); formal analysis (equal); investigation (equal); software (equal); writing – original draft (equal). **Feng Luo:** Supervision (equal); writing – review and editing (equal). **Xi Yan:** Conceptualization (equal); funding acquisition (equal); methodology (equal); project administration (equal); writing – review and editing (equal).

## FUNDING INFORMATION

None.

## CONFLICT OF INTEREST STATEMENT

No competing interests.

## Data Availability

The data used and/or analyzed during the study can be obtained from the corresponding author on reasonable request.
